# Burden of upper gastrointestinal cancers in the east of Golestan province (Golestan cohort study)

**DOI:** 10.1002/cnr2.2001

**Published:** 2024-03-04

**Authors:** Mohammad‐Ali Jahani, Raziyeh Esmaeili, Mahdi Abbasi, Hossein‐Ali Nikbakht, Habibollah Azarbakhsh, Gholamreza Roshandel, Sahar Delavari, Layla Shojaie, Ghahraman Mahmoudi

**Affiliations:** ^1^ Social Determinants of Health Research Center Health Research Institute, Babol University of Medical Sciences Babol Iran; ^2^ Health Services Management Golestan University of Medical Sciences Gorgan Iran; ^3^ Department of Health Economics and Management School of Public Health, Tehran University of Medical Sciences Tehran Iran; ^4^ Student Research Committee Shiraz University of Medical Sciences Shiraz Iran; ^5^ Golestan Research Center of Gastroenterology and Hepatology Golestan University of Medical Sciences Gorgan Iran; ^6^ Institute for the Developing Mind, Children's Hospital Los Angeles, Keck School of Medicine, University of Southern California Los Angeles California USA; ^7^ Division of GI/Liver, Department of Medicine Keck school of Medicine, University of Southern California Los Angeles California USA; ^8^ Hospital administration Research Center, Sari Branch, Islamic Azad University Sari Iran

**Keywords:** burden of disease, disability‐adjusted life years, Iran, upper gastrointestinal cancer

## Abstract

**Background:**

Cancers, especially Upper Gastrointestinal Cancers (UGCs), pose a substantial burden on society, particularly in developing nations. Golestan province, Iran, is known for its high UGC rates globally.

**Aims:**

This study delves into the disease burden of UGCs in the eastern part of Golestan province.

**Methods and Results:**

This study was conducted using the results of the Golestan cohort study. 2711 patients participating in this cohort, who visited Atrak Clinic during 2001–2020, participated in this study. After excluding patients with incomplete records, 2481 patients were included in the study. To compute the metrics of years of life lost (YLL), years of life lived with disability (YLD), and disability‐adjusted life years (DALY), we utilized the World Health Organization's standard life table, stratified by age and gender. The majority of UGC patients in our study were married (81.8%), had limited formal education (82.6%), and were predominantly male (61.1%). A substantial proportion resided in suburban areas (85.8%), and over half of the patients (52%) reported a history of drug addiction. The mean age at diagnosis for men was 65.76 years with a standard deviation of 11.34, while for women, it was 64.38 years with a standard deviation of 11.66. Regarding disease impact, YLL, YLD, and DALY for men were 21 240, 1956, and 23 196 (307.8 per 100 000), respectively. For women, these figures were 15 609 for YLL, 1367 for YLD, and 16 976 (223.1 per 100 000) for DALY.

**Conclusion:**

After the increasing trend of the burden of UGCs in Golestan province in the early years of the study, this rate has been decreasing in recent years. Effective strategies necessitate collaborative efforts across various sectors to alleviate this burden, focusing on preventive measures, timely diagnosis, and well‐coordinated therapeutic interventions.

## 1 INTRODUCTION

The global disease landscape has shifted toward non‐communicable diseases, with the primary drivers of this transformation being urbanization and demographic shifts within the population.^1^ Currently, non‐communicable diseases are the main cause of death worldwide.^2^ Cancers rank among the most prevalent non‐communicable diseases and stand as the leading cause of mortality in almost 50 high‐income nations. Notably, lung, liver, stomach, colorectal, breast, and cervical cancers feature prominently among the top ten causes of cancer‐related fatalities worldwide.^1^ In 2019, there were approximately 23.6 million newly diagnosed cases and 10 million cancer‐related deaths worldwide. Among the 22 groups of diseases and injuries in the global burden of disease study GBD 2019, cancer ranked second globally after cardiovascular diseases in terms of mortality, years of life lost (YLL), and disability‐adjusted life year (DALY).^3^

Statistics reveal that nearly three‐quarters of non‐communicable disease‐related mortalities occur in low‐ and middle‐income nations.^2^ For example, in the Eastern Mediterranean Region, about 60% of deaths are attributed to non‐communicable diseases.^4^ It is expected that this ratio will increase to 70% by 2030.^5^ Furthermore, the heightened prevalence of risk factors associated with non‐communicable diseases, including physical inactivity, obesity, tobacco use, and the consumption of foods rich in salt, sugar, and fat, has raised significant concerns in these regions.^4^ In Iran, the rise of non‐communicable diseases has evolved into a substantial challenge. In 2019, these diseases led to the loss of 508 326 lives, marking an 88% increase from the figures observed in 1990. The number of premature deaths attributed to the four primary non‐communicable diseases amounted to 66 818 in 1990, but by 2019, this number had surged significantly to 893 100 cases.^6^ Breast, colorectal and stomach cancers were the most prevelant cancers in Iran in 2016 and it is anticipated that they will remain the leading types of cancers in the country through 2025.^7^

Gastrointestinal cancers (GCs) reduce health‐related quality of life due to their aggressive nature and treatment complexity.^8^ Moreover, aligning with the objectives set forth in Sustainable Development Goal (SDG) 3.4, Iran is striving to reduce the percentage of premature deaths to 11.6% by 2025. However, this target appears challenging to attain given the current circumstances characterized by unhealthy lifestyles, a high disease burden, and inadequate investments in preventive measures, among other factors.^6^

Golestan province in northern Iran is known as a high‐risk area for GCs.^9^ Controlling the high incidence of upper gastrointestinal cancers (UGCs) in the northern regions of Iran requires the implementation of corrective interventions. These interventions should cover the areas of prevention, early detection, treatment, and palliative care.^10^ The design and implementation of these interventions requires the existence of effective evidence regarding the incidence, prevalence and burden of these cancers. Few studies have been conducted in Iran regarding the long‐term status of UGCs. This study was conducted with the aim of investigating the burden UGCs in Golestan province.

## 2 METHODS

This study was conducted using the data of the Golestan cohort. The Golestan cohort study was conducted in 1997 by the Digestive Diseases Research Institute (DDRI) of Tehran University of Medical Sciences. After that, in 2007, the main study of the Golestan cohort was conducted. The primary objective of this cohort was to identify the factors contributing to the high incidence of esophageal cancer among individuals aged 40–75 years in regions with a high prevalence of gastrointestinal tract cancers, specifically Gonbad and Kalaleh cities in Golestan province. The cohort comprised randomly selected individuals from both urban and rural areas within these two cities, with 10 032 residents from urban areas and 40 013 residents from rural areas registered for the study.^11^, ^12^

The data collected in Golestan cohort includes, demographic characteristic, lifestyle information, as well as biological samples such as blood, urine, hair, and nail specimens. Dietary habits were assessed using a specially tailored food frequency questionnaire (FFQ) that accounted for the region's distinct dietary patterns. Cohort participants receive monthly follow‐ups via phone calls. The study benefits from several factors that enhance its reliability, including the storage of biological samples in both Tehran and France, international collaborations with organizations such as the International Agency for Research on Cancer (IARC), the National Cancer Institute (NCI), and Cambridge University, the establishment of a specialized clinic (Atrak Clinic), adherence to international standards for biological sample preservation, a broad population coverage, and a lengthy follow‐up period for study participants.^11^

Every participant in the Golestan cohort was issued an ID card upon registration. This ID card served as a means for them to seek medical attention at Atrak Clinic in case they experienced digestive symptoms. Atrak Clinic, situated within the primary hospital in Gonbad city, is a specialized gastroenterology clinic established by the Digestive Diseases Research Institute (DDRI). At this clinic, individuals undergo health assessments, and their medical records are kept in the registration system. Furthermore, they receive diagnostic and treatment services at no cost. For all cohort participants who visited Atrak Clinic, personalized files were established, encompassing comprehensive information pertinent to their health and medical history. Monthly, the databases at Atrak Clinic and the Cancer Registration Center in Golestan province were cross‐referenced to identify any instances of cancer among the individuals participating in the cohort.^11^

In this current study, we utilized the data contained within the patient files for individuals diagnosed with upper gastrointestinal cancers, which includes esophageal and stomach cancers, spanning the years 2001–2020. A total of 2711 patient cases with UGCs were documented and registered at Atrak Clinic. The selection of all these patients was based on a complete census. Following the exclusion of individuals with incomplete records, we utilized the patient records of 2481 cases. The extraction of patient information from their files was carried out using an Excel‐based data extraction form.

### 2.1 DALY

DALYs provide a comprehensive measure of the total time lost due to a specific health condition, encompassing both premature death and disability. Originally developed for the World Health Organization's Global Burden of Disease (GBD) study in 1990, DALYs have since evolved into widely accepted standard metric for quantifying the burden of various diseases.^13^

We calculated the DALY for cancer using procedures derived from those described in the GBD study, which summed the YLL and YLD components. The basic formula is expressed as follows^14^:

DALY=YLL+YLD.

To calculate the YLL, YLD and DALY, the standard life table of the WHO was used for different age and sex groups. Based on this table, the life expectancy for men was considered equal to 80 years, and for women, 82.5 years. The following formula calculates the YLL.^15^

YLL=NL.

In this formula, N is equal to the number of deaths due to UGCs in a certain age and sex group, and L is equal to the standard life expectancy of deceased people of the same age and sex.^16^

The mean age was used to calculate YLD. The disability weight was estimated using the items described in the GBD and was considered 0.544.^13^ The duration of the disease was considered from the time of diagnosis to the time of conducting the study. The following formula was used to calculate YLD.^15^

$$\mathrm{YLD}=\text{Incidence}\times \text{Duration}\times \mathrm{DW}\;\left(\text{Disability weight}\right).$$

And T‐test and analysis of variance were used to compare mean YLL, YLD, and DALY in different age and sex groups.

Patients' data were exported SPSS version 25 software from Excel software. Mean, standard deviation, frequency, and percentage were used to describe quantitative data.

## 3 RESULTS

The majority of patients with UGC were married, had limited formal education, were male, resided in rural areas, and lacked a family history of the disease. Additionally, a significant proportion of the identified patients belonged to the Turkmen ethnic group, and over half of them had a history of drug addiction (Table 1).

**TABLE 1:** Patients demographic with UGCs between 2001 and 2020 in Golestan cohort.

Variable		Frequency	%	Variable		Frequency	%
Marital status	Married	2029	81.8	Educational level	No schooling	2050	82.6
	Widow	420	16.9		Less than a diploma	370	14.9
	Single	27	1.9		Diploma	37	1.5
	Divorced	5	2		University/college	24	1
City of residence	Gonbad Kavous	1105	44.5	Ethnicity	Turkmen	1358	54.7
	Kalale	573	23.1		Persian	529	21.3
	Minoodasht	450	18.1		Persian	270	10.9
	Azadshahr	203	8.2		Sistani	207	8.3
	Ramian	150	6.1		Baloch	73	2.9
Sex	Male	1517	61.1		Other	44	1.8
	Female	964	38.9	Place of residence	Rural	2128	85.8
Age	<35 years old	25	1		Urban	353	14.2
	35–44 years old	84	3.4	Family history	Yes	569	22.9
	45–54 years old	330	13.3		No	1912	77.1
	55–64 years old	668	26.9	Job	Farmer	991	39.9
	65–74 years old	809	32.6		Housekeeper	931	37.5
	75–84 years old	508	20.5		Self‐Employment	358	14.4
	>85 years old	57	2.3		Retired	172	6.9
History of addiction	Opium	891	35.9		Employee	29	1.2
	Cigarettes	479	19.3	Type of cancer	Squamous cell carcinoma (SCC)	1341	54.1
	Naswar	291	11.7		Adenocarcinoma (ADC)	1033	41.6
	Hookah	122	4.9		SCC and ADC	13	0.5
	Alcohol	49	19.7		Carcinoma	94	3.8

**FIGURE 1:**
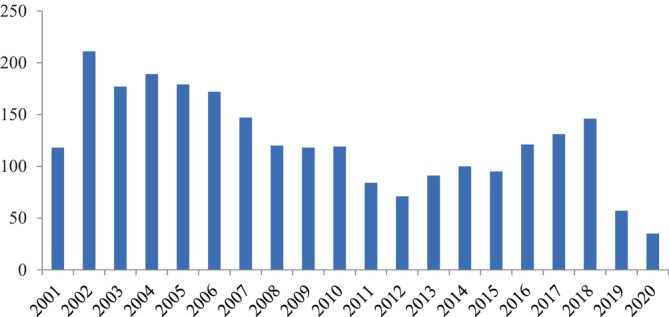
The number of patients with UGCs in the studied population from 2001 to 2020 in Golestan cohort.

The number of patients with UGCs was 118 in 2001, which reached 57 in 2019. The highest number of patients identified was in 2002 (Figure 1).

**FIGURE 2:**
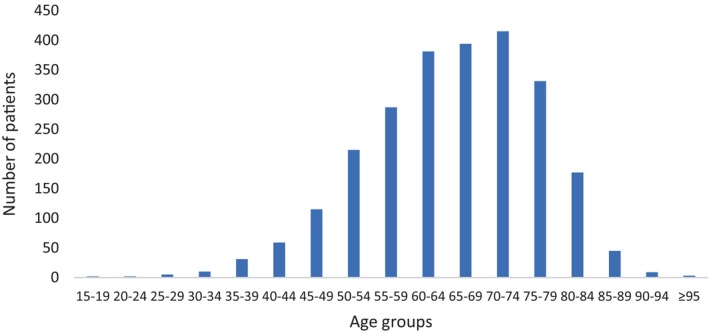
Age distribution of UGCs in the studied population between 2001 and 2020 in Golestan cohort.

The disease onset age, was considered equivalent to the age when gastrointestinal symptoms, particularly dysphagia, first appeared. The average disease onset age for men was 65.76 ± 11.34, while for women, it was 64.38 ± 11.66. (Figure 2).

Total number of DALY due to UGCs in the study population was 40 172 (265.2 per 100 000) years (Table 2).

**TABLE 2:** The total number of YLL, YLD and DALYs caused by UGCs in the studied population “between 2001 and 2020 in Golestan cohort”.

Year	YLL		YLD		DALYs		DALYs (per 100 000)	
	Male	Female	Male	Female	Male	Female	Male	Female
2002	1358	1220	101	54	1459	1274	427.4	360.9
2003	2000	1751	44	81	2044	1832	587.6	510.7
2004	1580	1188	122	83	1702	1271	480.3	348.7
2005	1846	1515	208	209	2054	1724	569.2	465.6
2006	1023	806	350	194	1373	1000	373.8	265.9
2007	1282	1111	180	140	1462	1251	391.1	327.6
2008	856	650	205	101	1061	751	279.0	193.7
2009	950	554	144	65	1094	619	282.8	157.3
2010	969	590	136	69	1105	659	280.9	165.1
2011	809	503	51	33	860	536	215.1	132.4
2012	1055	703	52	39	1107	742	273.3	181.6
2013	631	360	50	55	681	415	166.0	100.7
2014	1089	571	50	37	1139	608	274.2	146.2
2015	519	647	53	38	572	685	136.0	163.3
2016	1065	698	61	48	1126	746	264.5	176.3
2017	1143	786	59	34	1202	820	279.1	191.6
2018	1365	954	50	53	1415	1007	324.8	232.6
2019	890	463	30	27	920	490	208.8	111.9
2020	810	539	12	8	822	547	184.4	123.5
Total	21 240	15 609	1956	1367	23 196	16 976	307.8	223.1
*p* value	.021		.189		.019		.046	

**FIGURE 3:**
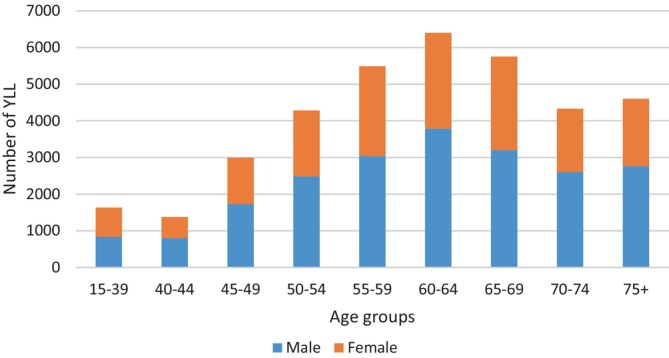
Number of YLL stratified by age groups and sex caused by UGCs in the studied population between 2001 and 2020 in Golestan cohort.

The highest and lowest number of YLL were in the age groups of 60–64 years and 40–44 years, respectively (Figure 3).

**FIGURE 4:**
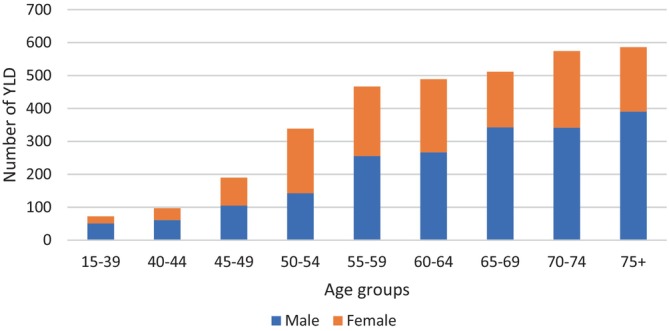
Number of YLD stratified by age groups and sex caused by UGCs in the studied population between 2001 and 2020 in Golestan cohort.

The highest number of YLD in men and women were in the age groups above 75 and 70–74, respectively (Figure 4).

**FIGURE 5:**
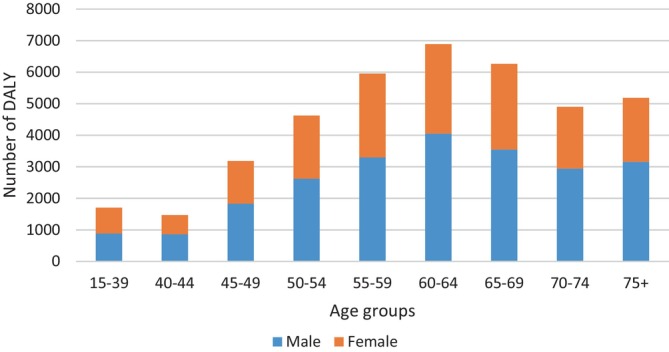
Number of DALY stratified by age groups and sex caused by UGCs in the studied population between 2001 and 2020 in Golestan cohort.

The highest and lowest DALY in both sexes were in the age groups of 60–64 years and 40–44 years, respectively (Figure 5).

A statistically significant difference exists in the means of the YLL and DALY indexes between males and females, although no significant difference was observed for the YLD index (Table 2). Furthermore, a statistically significant difference in the means of YLL (*p*‐value = .038), YLD (*p*‐value = .033), and DALY (*p*‐value = .030) indexes was noted across different age groups.

## 4 DISCUSSION

This study aimed to assess the burden of UGCs in the eastern region of Golestan province, Iran. Among the 2481 patients with UGCs, most were male, married, had limited formal education, resided in rural areas, lacked a family history of the disease, had a history of addiction, and were in the age range of 65–74 years.

The study revealed a total of 40 172 (265.2 per 100 000) DALYs within the study population. Out of these, 23 196 (307.8 per 100 000) DALYs were attributed to men, and 16 976 (223.1 per 100 000) DALYs were related to women. Additionally, the study found 36 849 YLL and 3323 YLD. The male‐to‐female sex ratio for DALYs was 1.36, indicating a higher burden of disease among men.

In 2017, there were 19.1 million deaths globally attributed to gastric cancer, and this number increased to 22.2 million by 2019. Furthermore, the age‐standardized DALY rate, per 100 000 population, was 235.9 in 2017. In 2020, the worldwide incidence of gastric cancer was 11.3 cases per 100 000 people, and for esophageal cancer, it was 6.3 cases per 100 000 people, resulting in 1 089 103 new cases of gastric cancer and 604 127 new cases of esophageal cancer.^17^, ^18^ In recent years, the incidence of UGCs has decreased significantly. For example, in a study between 1997 and 2017, the incidence of gastric cancer decreased by more than 47%.^18^ This downward trend is observed in many countries worldwide.^19^

In Iran, previous studies have reported a range of incidence rates for gastric cancer, spanning from as low as 0.1 to as high as 26 cases per 100 000 people. For esophageal cancer, the incidence rates have been documented to vary between 4.6 and over 50 cases per 100 000 people.^20^, ^21^, ^22^, ^23^, ^24^ The northern regions of Iran, particularly Golestan province, have consistently exhibited a high prevalence of UGCs. Within this province, the disease has demonstrated higher prevalence rates, particularly among the ethnic Turkmen population residing in areas like Gonbad Kavous and Kalaleh.^23^ In Ghasemi‐Kebria et al.'s study, the age‐standardized incidence rates (ASRs) of gastric cancer in this province in 2016 in men and women were 26.9 and 12.2 per 100 000 people, respectively.^22^ This rate for esophageal cancer in this province between 2004 and 2008 was equal to 24.3 in males and 1.19 in females.^24^

In the present study, the DALY attributed to UGCs in the studied population of Golestan province was 40 172. According to Wang et al.'s study, the standardized rate of gastric cancer in China in 2016 was 464.47 per 100 000 people.^25^ In Song et al.'s study, this rate was 268.4 for the whole world in 2019. Also, in this study, the number of deaths attributed to gastric cancer worldwide was estimated at 22.2 million.^26^ Iran and China have a high burden of DALY attributed to UGCs. Wong et al.'s research has highlighted the prevalence of UGCs in the Asian belt, which includes northern Iran, Turkey, Central Asia, and northern and central China, as consistently having one of the highest incidence rates globally.^27^

The findings from the Golestan cohort study have indicated that several risk factors contribute to UGCs. These include an inadequate diet, low socioeconomic status, thermal damage from consuming hot tea, and exposure to carcinogens associated with opium use.^11^ In their study, Gupta et al. showed that most cancer‐related DALYs occur among individuals with lower economic and social status.^28^ Fitzmaurice et al. highlighted the inadequacies within the healthcare systems of developing countries in delivering care for non‐communicable diseases as a contributing factor to the elevated burden of cancer in these nations.^29^

Li et al. argued that UGCs disproportionately affect economically disadvantaged and socially marginalized individuals, primarily because of reduced access to healthcare and education, along with poor living conditions.^30^ Kumar et al. conducted an analysis of global trends in UGCs and found that a significant proportion, specifically three‐quarters, of these cancers occurred in developing countries in the year 2020.^17^ Several studies showed the most important risk factors for UGCs in Iran, include drug abuse, alcohol consumption, exposure to environmental pollutants and chemicals, Helicobacter pylori infection, economic and social status, family history of the disease, dietary habits, and age.^11^, ^24^, ^31^

In the present study, more than 82% of patients were illiterate. Zarea and colleagues believed that illiterate people are unaware of the importance of annual screening and check‐ups. onsequently, they tend to undergo fewer screening tests and are often diagnosed at advanced stages of the disease.^32^ In their study, Goding Sauer et al. investigated cancer risk factors in the United States and found that individuals with lower levels of education have a higher prevalence of modifiable risk factors for cancer compared to those with higher education levels. Furthermore, their research demonstrated that individuals with lower educational attainment are less likely to undergo cancer risk screening. For instance, nearly half of women without a college degree were obese, while only one‐third of college graduates were obese. This highlights disparities in risk factors and screening behavior related to education level.^33^

Other research results showed that more than 61% of patients are men. This result was similar to previous studies of UGCs in Iran, where the gender ratio of cancer shown a higher incidence among men compared to women.^34^, ^35^ For example, in the study of Farmanfarma et al. in 2020, the incidence of gastric cancer in men is 74.9 per 100 000 people, while in women, this rate is equal to 4.6 per 100 000 people.^31^ Arnold et al also showed in their study in 2020 that globally, gastrointestinal cancers are twice as common in men as in women.^36^ Men tend to have greater exposure to environmental pollutants, a higher prevalence of behavioral risk factors, and may receive less preventive care, all of which could contribute to this trend. However, Shadmani et al. showed that while the overall mortality rate in men has been declining in recent years and will remain stable until 2030, it will increase among women in this time frames.^37^ Therefore, it is necessary to implement interventions focusing on training and raising public awareness among women, emphasizing the importance of annual screening and adopting strategies to improve access to screening tests and healthcare facilities.

In this study, more than 82% of the examined patients were over 55 years old. Also, more than 55% of patients were 65 years old and older. These findings align with the results from Zarea et al.'s study, which demonstrated that the prevalence of gastrointestinal cancer is highest among individuals aged 65 years and older.^32^ Lin et al.'s study in 2022 showed that the risk of UGCs gradually increases with age.^19^ A study in China showed that people between 65 and 69 years of age are at relatively high risk for UGCs.^30^ In another study in Rwanda, the average age of esophageal and stomach cancer patients was 54.9 and 56.9 years among men and women, respectively.^38^ These results underscore the importance of prioritizing and promoting annual screenings, especially for individuals aged 50 and above.

Putting greater emphasis on screening, especially for individuals with risk factors, is essential. Financial support measures, such as insurance coverage and subsidies, should be considered, particularly for those in lower socioeconomic brackets. Furthermore, the role of primary healthcare providers in educating and empowering middle‐aged and elderly individuals about the importance of screening cannot be understated.

A substantial portion of the global cancer burden is linked to modifiable risk factors. Based on this, it is necessary to carry out corrective interventions to reduce the population's exposure to modifiable risk factors. Furthermore, there exist non‐modifiable risk factors, including age, genetic predisposition, and family history, which significantly influence the onset of this disease. In such instances, it is essential to prioritize interventions geared toward early detection and effective treatment. In addition, due to the increase in the survival of patients with UGCs in recent years and the high complications of surgical procedures, it is necessary to focus more on multimodal interventions to minimize the adverse effects of cancer treatments and increase the quality of life of survivors.

This study has notable strengths, including its extensive study duration and the use of highly reliable information. However, there are several limitations that should be acknowledged. Firstly, the study lacks information concerning the stage of UGC, the distribution of treatment modalities, and survival outcomes. Moreover, a key limitation is that the study only included the population of two specific cities, which may impact the generalizability of its findings. Caution should be exercised when extending the results to broader populations.

## 5 CONCLUSION

This study has revealed that between 2001 and 2020. Approximately 45% of the patients under examination were in the age group of economic activity, which is typically below 65 years old. This demographic could potentially impose substantial economic costs on the country, both through healthcare expenditures and household expenses related to treatment. Furthermore, over 85% of the patients resided in rural areas, where unhealthy dietary habits and lifestyles, such as the consumption of hot tea, drug use, and certain detrimental dietary practices like smoked fish consumption, appear to be more prevalent. Consequently, there is a clear need to place greater emphasis on interventions aimed at empowering the population and prioritizing primary prevention measures.

## AUTHOR CONTRIBUTIONS

**Mohammad‐Ali Jahani**: Project administration (equal); Writing – review & editing (equal). **Raziyeh Esmaeili**: Conceptualization (lead); Resources (equal); Data curation (equal). **Mahdi Abbasi**: Writing – review & editing (lead). **Hossein‐Ali Nikbakht**: Data curation (equal); Formal analysis (equal); Methodology (equal). **Habibollah Azarbakhsh**: Methodology (equal); Formal analysis (equal). **Gholamreza Roshandel**: Data curation (equal); Resources (equal). **Sahar Delavari**: Writing – review & editing (equal). **Layla Shojaie**: Writing – review & editing (equal). **Ghahraman Mahmoudi**: Project administration (equal); Resources (equal); Methodology (equal).

## CONFLICT OF INTEREST STATEMENT

The authors have stated explicitly that there are no conflicts of interest in connection with this article.

### ETHICS STATEMENT

In this study, to maintain the confidentiality of the information, the code and record number were used instead of the names of the patients. Also, necessary agreements were obtained to receive patient information.

## Data Availability

The data that support the findings of this study are available from the corresponding author upon reasonable request.
